# Safety and efficacy of adjuvant Sotagliflozin therapy in patients with T1D - an update and systematic review and meta-analysis

**DOI:** 10.3389/fendo.2025.1506652

**Published:** 2025-06-03

**Authors:** Yunzhen Lei, Shanshan Yao, Zhenglong Wang, Qianxian Tu, Zhengqiang Yuan, Qianfeng Jiang

**Affiliations:** ^1^ Department of Clinical Medicine, Zunyi Medical University, Zunyi, Guizhou, China; ^2^ Department of Cardiovascular Medicine, The Third Affiliated Hospital of Zunyi Medical University, Zunyi, China

**Keywords:** Sotagliflozin, type 1 diabetes, SGLT1, SGLT2, meta-analysis

## Abstract

**Objective:**

This meta-analysis aims to assess the safety and efficacy of Sotagliflozin in patients with type 1 diabetes (T1D).

**Methods:**

Data on target organ protection, blood glucose levels, blood pressure, weight, insulin usage, and adverse events (AEs) associated with Sotagliflozin in the treatment of T1D were collected from databases including PubMed, Scopus, Web of Science, Embase, and the Cochrane Library. The search period extended until February 21, 2024, and included studies were restricted to randomized controlled trials (RCTs) investigating Sotagliflozin for T1D. The meta-analysis was performed using Stata 14 and RevMan 5.4.

**Results:**

A total of 12 randomized controlled trials were included in the analysis, with treatment durations ranging from 14 to 52 weeks. Sotagliflozin, when used in combination with insulin therapy, resulted in significant reductions in cardiovascular disease (CVD) risk (−6.38%; *95% CI:* −7.63 to −5.1; *P* < 0.05) and end-stage kidney disease (ESKD) risk (−5.0%; *95% CI:* −7.62 to −2.3; *P* < 0.05). Additionally, Sotagliflozin significantly reduced blood glucose, blood pressure, and body weight, with these effects showing dose- and duration-dependent trends. Regarding adverse effects, the combination of insulin and Sotagliflozin was associated with an increased incidence of genital infections (Sotagliflozin group: 8% vs. control: 2%) but a reduced risk of fractures (Sotagliflozin group: 1% vs. control: 2%). No statistically significant differences were observed between the two groups for other outcomes, including diabetic ketoacidosis (DKA), hypoglycemia, mortality, cancer, nausea, diarrhea, urinary tract infections, or liver and kidney function impairment.

**Conclusion:**

In T1D patients, Sotagliflozin adjunct therapy improves blood glycemia, stabilizes blood pressure, and reduces cardiovascular risk factors. It also shows potential in lowering fracture risk, but the risk of DKA requires further clinical validation.

**Systematic review registration:**

https://www.crd.york.ac.uk/PROSPERO/#joinuppage, identifier CRD42023467427.

## 1 Introduction

Diabetes mellitus comprises two primary types: type 1 diabetes (T1D) and type 2 diabetes (T2D) (1). T1D, characterized by autoimmune-mediated pancreatic beta-cell destruction, leading to exclusive dependence on insulin therapy for management (2). However, this approach often leads to suboptimal glycemia with marked fluctuations, thereby increasing the risk of target-organ damage. Although sodium-glucose cotransporter 2 (SGLT2) inhibitors demonstrate partial efficacy in preserving target organs and improving glycemia, their use in T1D patients is associated with a higher incidence of adverse events (AEs) (3), particularly diabetic ketoacidosis (DKA), which contributes to elevated mortality rates in T1D (4, 5).

Sotagliflozin, a dual inhibitor of sodium-glucose cotransporters 1 and 2 (SGLT1/2), combines the advantages of SGLT2 inhibitors (6), such as improved glycemia, reduced cardiovascular adverse events, and enhanced survival benefits. Concurrently, SGLT1 inhibition mitigates side effects associated with SGLT2 inhibitors, including a lower incidence of DKA, urinary tract infections, and improved acid-base buffering capacity (7, 8). However, clinical randomized controlled trials (RCTs) have reported inconsistent findings, most notably an elevated risk of DKA, which remains contradictory and controversial (9, 10). Thus, this drug shows significant potential for T1D treatment if supported by robust evidence. To address this gap, we conducted an updated systematic review and meta-analysis with the following objectives: 1). Inclusion of high-quality RCTs to strengthen result reliability; 2). Comprehensive assessment of efficacy and safety, including target-organ protection, estimated glomerular filtration rate (eGFR), fracture rates, and major adverse cardiovascular events (MACE); 3). Subgroup analyses and meta-regression were conducted to explore correlations between outcomes, treatment duration, and drug dosage, thereby reinforcing the evidence for Sotagliflozin’s safety and efficacy as an adjuvant therapy in T1D; 4. Comparative evaluation of current meta-analysis findings.

## 2 Materials and methods

### 2.1 Protocol

This systematic review and meta-analysis strictly adhered to the protocol registered with PROSPERO (CRD42023467427) and followed the guidelines outlined in the PRISMA statement.

### 2.2 Search crit

#### 2.2.1 Inclusion criteria eria

The study design adhered to the PICOS framework: (1) Population (P): Patients diagnosed with T1D. (2) Intervention (I): Administration of Sotagliflozin. (3) Comparison (C): The control group comprising patients with T1D managed exclusively with insulin therapy. (4) Outcome Measures (O): Evaluation of cardiovascular disease (CVD) risk, end-stage kidney disease (ESKD), fasting plasma glucose (FPG), 2-hour postprandial plasma glucose (2H-PPG), glycosylated hemoglobin (HbA1c) levels, basal insulin usage, bolus insulin usage, total insulin consumption, systolic blood pressure (SBP), diastolic blood pressure (DBP), estimated eGFR, body weight (Bw), and monitoring of common adverse effects. (5) Study Type (S):Randomized Controlled Trials (RCTs).

#### 2.2.2 Exclusion criteria

(1) Animal experiments,(2) Reviews and case reports,(3) Direct data from non-articles,(4) Duplicate published papers,(5) Patients with T1D treated with other medications.

### 2.3 Search databases

PubMed, Scopus, Web of Science, Embase, and Cochrane Library were searched from their establishment to February 21, 2024. The search strategy is shown in **Supplementary Data Sheet 1**.

### 2.4 Search strategy, data extraction, and quality assessments

**Figure 1:**
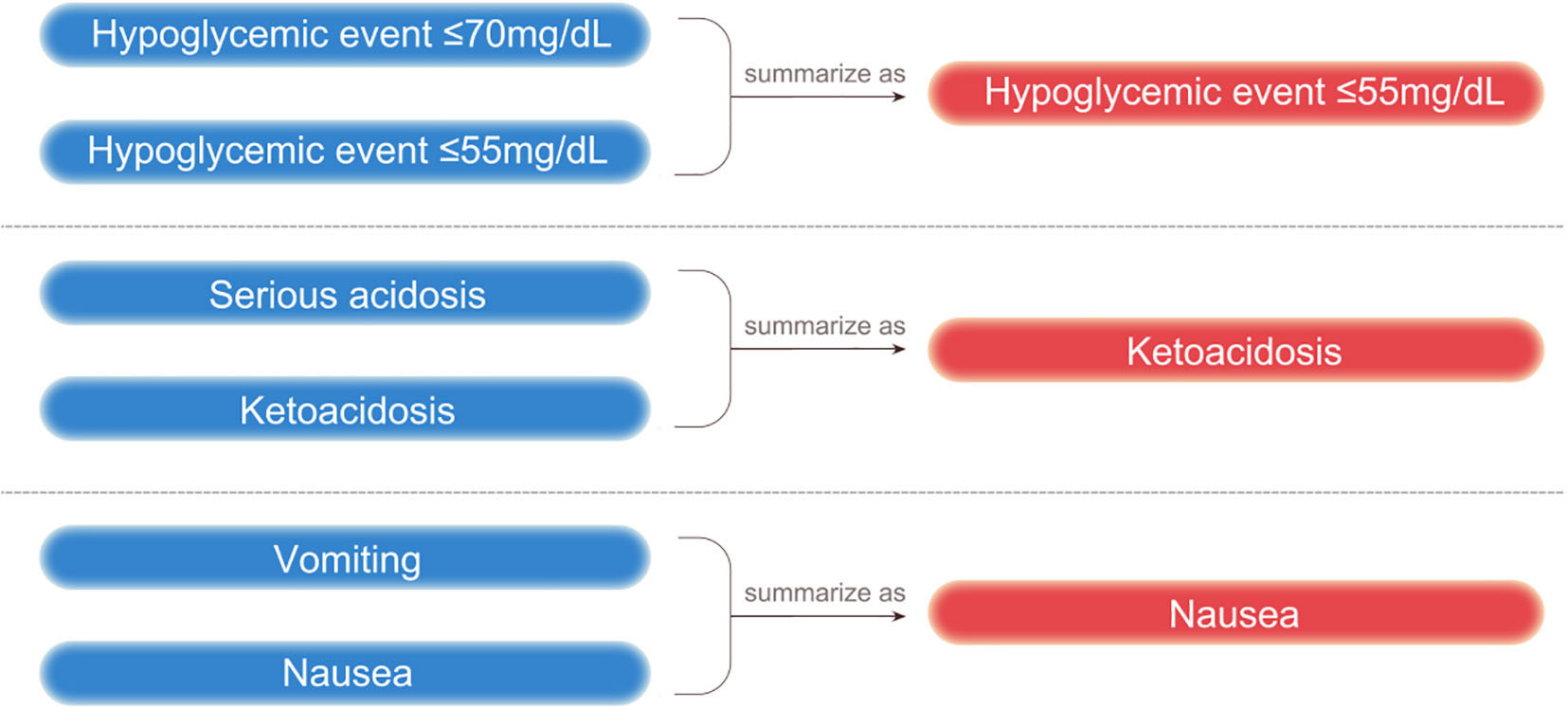
Infographic summarizing the categorization of indicators.

Two independent researchers conducted literature screening and data extraction in accordance with established inclusion and exclusion criteria. Initially, titles and abstracts were reviewed, and any articles that did not meet the inclusion criteria were excluded. The remaining articles underwent full-text review to determine their final eligibility. In cases of disagreement, consensus was reached through discussion among all researchers. Two researchers evaluated the eligibility of RCTs using a bias assessment tool to assess the quality of the literature. This tool considered randomization, allocation concealment, blinding, completeness of outcome data, selective reporting, and other potential sources of bias. Disagreements in the assessment were resolved through group discussion. Subsequently, reorganization and classification of the limited number of included studies were performed to mitigate publication bias. Further details of this process are provided in **Figure 1**.

### 2.5 Statistical analysis

The meta-analysis was performed using Stata 14.0 and RevMan 5.4 software. For efficacy outcomes, the extracted data represent the change from baseline to post-treatment period. For safety outcomes, the total number of adverse events in both groups was recorded. A continuity correction (e.g., Bartlett’s adjustment) was applied when AE incidence was 0% or 100%. Statistical heterogeneity among studies was evaluated using the *Q*-test and *I²* statistic, with heterogeneity defined as low (*I²* < 50%) or high (*I²* ≥ 50%). A fixed-effects model was used in the absence of significant heterogeneity, whereas a random-effects model was applied when heterogeneity was detected. For outcomes with high heterogeneity, sensitivity analyses were conducted to identify potential sources. Meta-regression explored variable correlations, and publication bias was assessed via Egger’s test, with *P* < 0.05 indicating potential bias. When bias was identified, the trim-and-fill method was used for adjustment. All outcomes were graded using the GRADE framework (**Supplementary Data Sheet 2**).

## 3 Results

### 3.1 Literature search results

**Figure 2:**
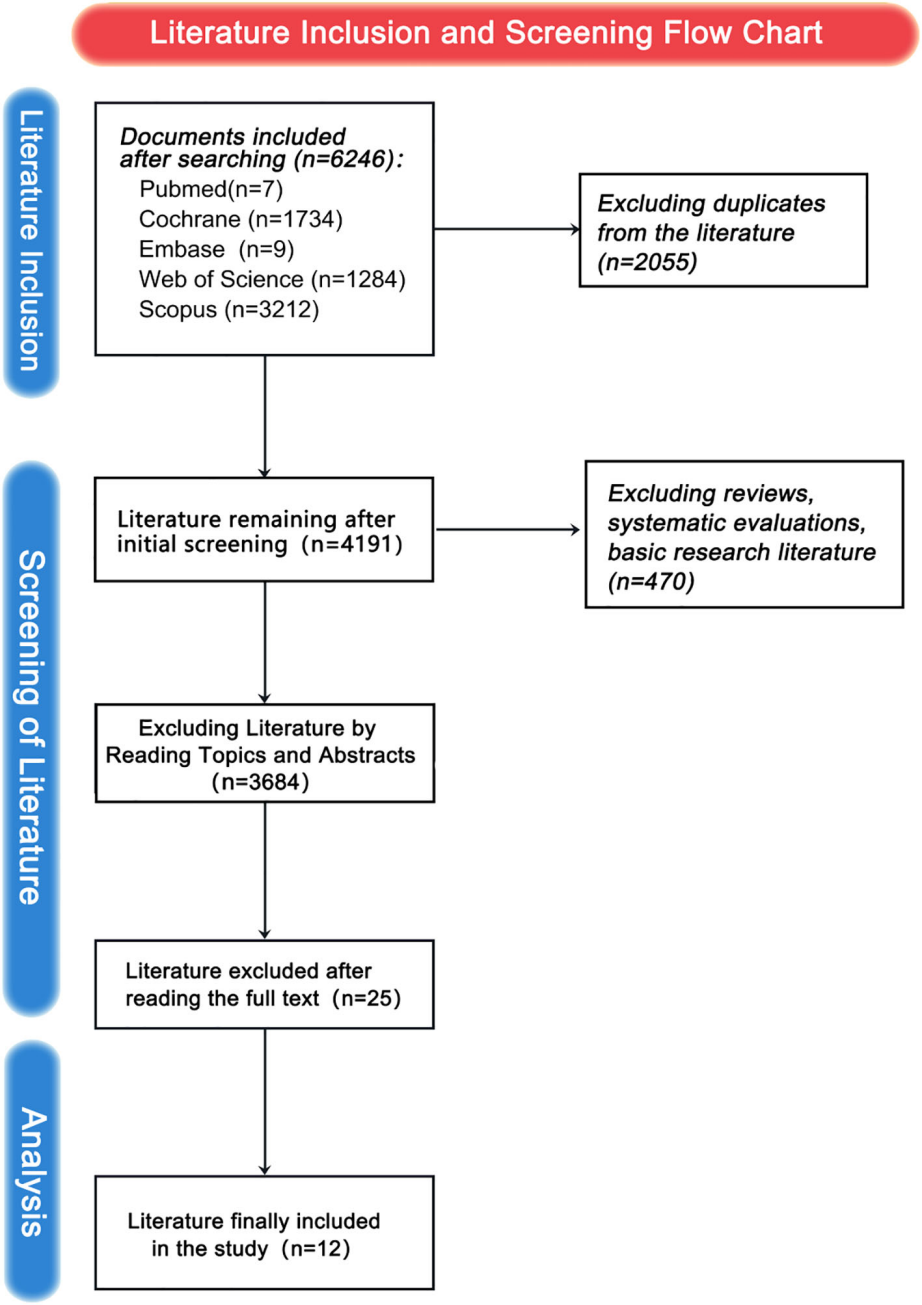
Process flowchart of article filtering.

In this study, an initial search retrieved 6,246 articles. After removing 2,055 duplicates, 37 unique articles remained for the first screening. Among them, 25 articles were excluded because they did not meet the inclusion criteria. As a result, 12 articles were retained for the final analysis. A visual representation of the literature screening process and its outcomes is presented in **Figure 2**.

### 3.2 Description of included trials

All of the studies were randomized controlled trials that examined a range of clinical parameters, including CVD, ESKD, FPG, 2H-PPG, HbA1c, basal insulin, bolus insulin, total insulin dosage, SBP, DBP, eGFR, body weight (Bw), DKA, adverse events, and serious adverse events. Detailed characteristics of these studies are provided in **Supplementary Data Sheet 3**.

### 3.3 Risk of bias assessments

**Figure 3:**
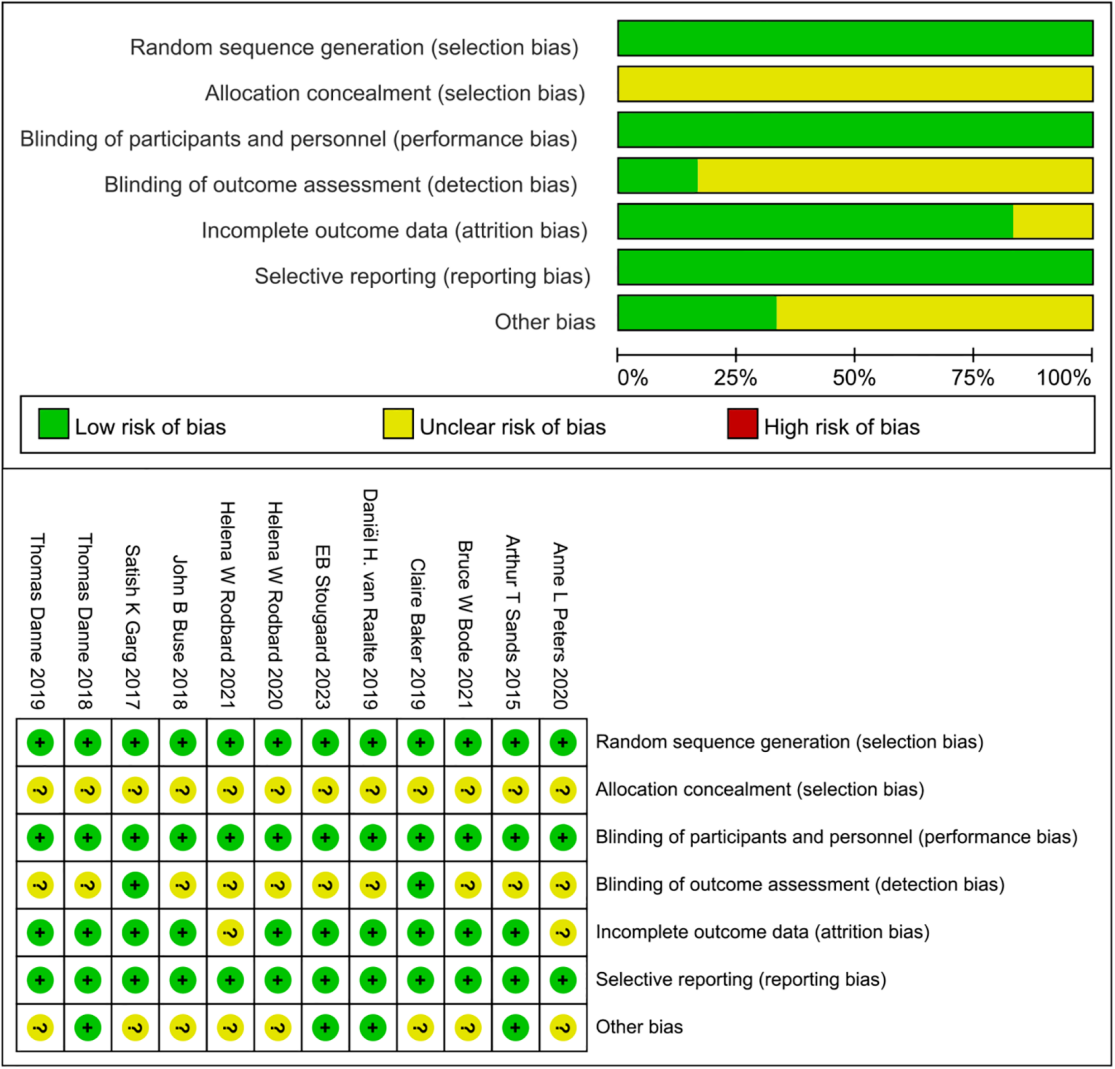
Quality evaluation chart of the literature.

All articles employed a randomized double-masked allocation method. However, it is important to note that the articles did not consistently clarify whether the statistical results underwent blinding procedures. To assess data reliability, the level of detail provided in the articles regarding patient follow-up and the recording of missed visits was crucial. The presence of selection bias depended on whether the articles explicitly defined specific population subgroups. Additionally, articles funded by public universities or charitable organizations were considered to have a low risk of other biases. The evaluations of treatment outcomes for each article are graphically depicted in **Figure 3**.

### 3.4 Target organ protection

**Figure 4:**
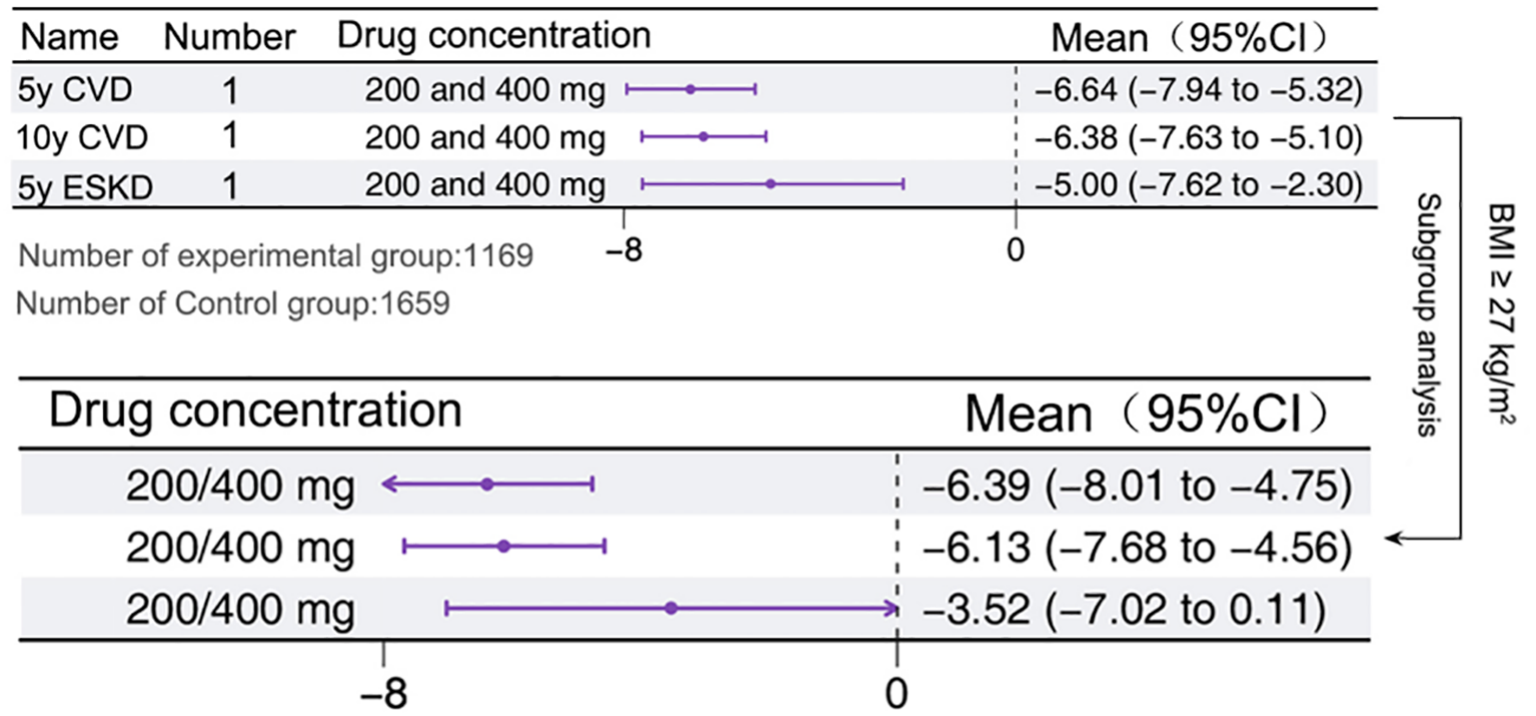
Protection of target organs in T1D patients treated with adjunctive therapy using Sotagliflozin.

A total of 1 study reported this outcome (11), with 1 arm, found that Sotagliflozin significantly reduced the likelihood of CVD [-6.38%, 95% CI: -7.63 to -5.1, P < 0.05] and ESKD [-5.0%, 95% CI: -7.62 to -2.3, P < 0.05] in T1D patients. However, a subgroup analysis of patients with a BMI ≥ 27 kg/m² revealed that the mitigating effect of Sotagliflozin on ESKD was significantly attenuated and no longer statistically significant. Nevertheless, it retained a substantial protective effect against CVD development (**Figure 4**).

### 3.5 Glucose regulation

**Figure 5:**
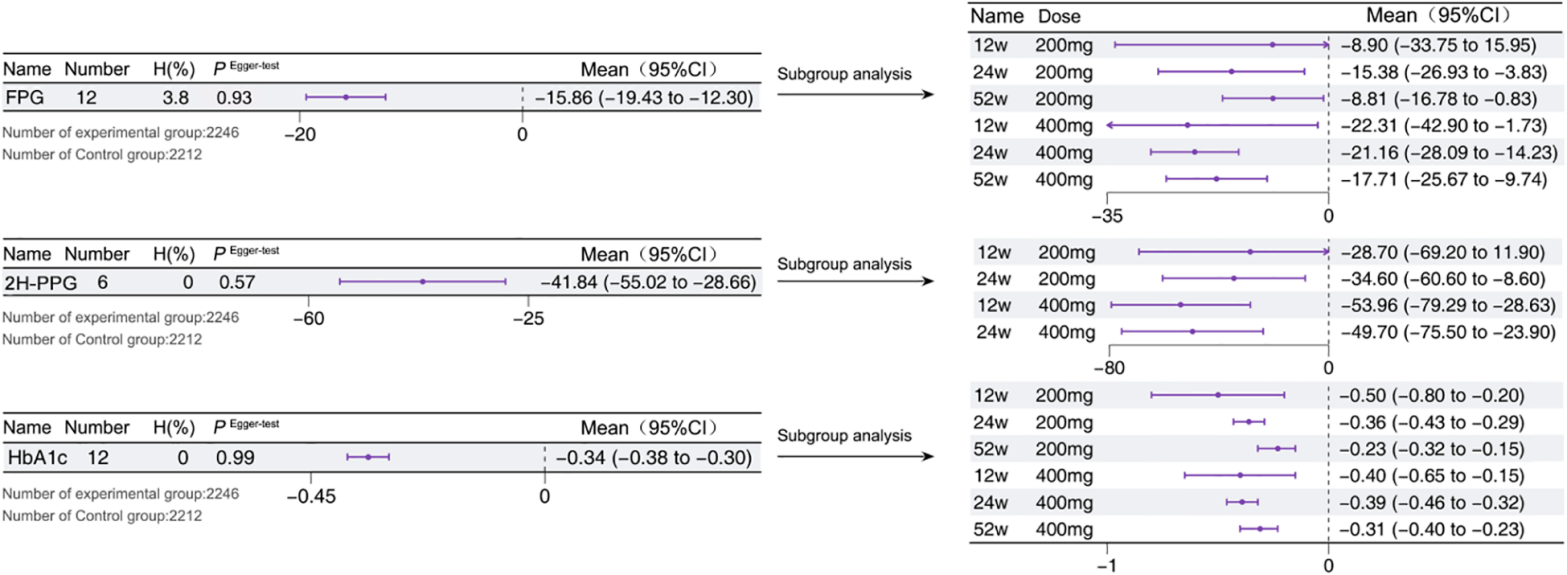
Glycemia in T1D patients treated with adjunctive therapy using Sotagliflozin.

A total of 4 studies reported on FPG (12–15), with a total of 12 arms. The results showed that adding Sotagliflozin to insulin therapy resulted in a significant reduction in FPG compared to insulin therapy alone [-15.86 mg/dL, *95% CI*: -19.43 to -23.30, *P* < 0.05] (*I^2^ =* 3.8, *P*>0.05). A total of 3 studies reported on 2H-PPG (12, 13, 16), with a total of 6 arms. The results indicated that the combination of Sotagliflozin and insulin therapy led to a substantial reduction in 2H-PPG [-41.84 mg/dL, *95% CI*: -55.02 to -28.66, *P* < 0.05] (*I^2^ =* 0.0, *P*>0.05). Subgroup analyses further revealed a positive correlation between drug concentration and the extent of 2H-PPG reduction within the 200 - 400 mg/day dose range. However, as the intervention duration increased, the reduction in blood glucose became more moderate, a trend consistent with the findings for HbA1c (12–15). All glycemic evaluation indices underwent Egger’s test to assess publication bias, and **Figure 5** visually presents the results.

### 3.6 Usage of insulin

**Figure 6:**
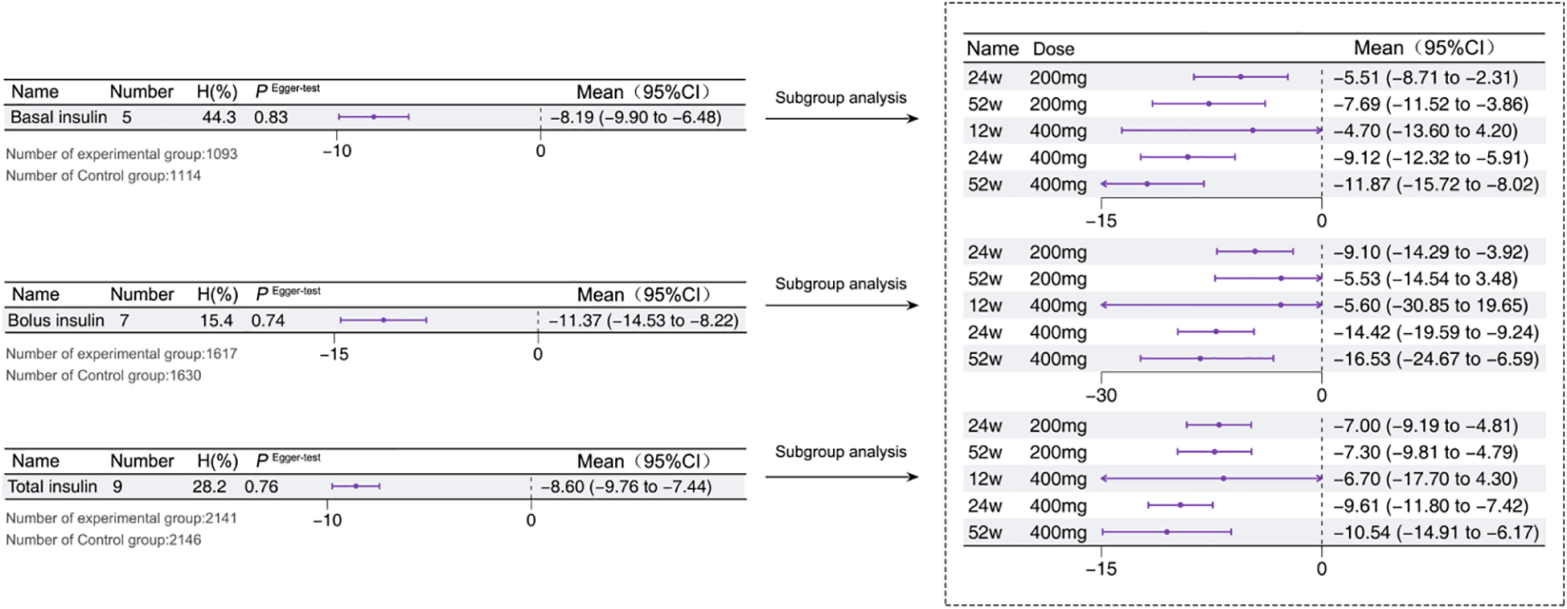
Insulin use in T1D patients treated with adjunctive therapy using Sotagliflozin.

A total of 2 studies reported this outcome (12, 15), with a total of 5 arms. The results demonstrated that the addition of Sotagliflozin to insulin therapy resulted in a significant reduction in basal insulin requirements compared to insulin therapy alone [-8.19%, *95% CI*: -9.90 to -6.48, *P* < 0.01] (*I^2^ =* 44.3, *P*>0.05). Subgroup analyses revealed a consistent trend of reduced basal insulin dosage, a pattern also observed with bolus insulin as the duration of Sotagliflozin intervention increased and the drug concentration escalated. Additionally, A total of 3 studies reported total insulin requirements (12, 14, 15), with a total of 9 arms. The results showed that Sotagliflozin combined with insulin therapy led to a significant reduction in total insulin requirements [-8.6%, *95% CI*: -9.76 to -7.44, *P* < 0.01] (*I^2^ =* 28.2, *P*>0.05). Subgroup analyses highlighted a consistent, significant decrease in total insulin usage with increasing drug concentration and longer intervention duration. All glycemic evaluation metrics underwent Egger’s test to assess publication bias, and **Figure 6** visually presents the results, confirming the absence of significant bias.

### 3.7 Continuous glucose monitoring time analysis

A total of 2 studies reported the outcome (12, 16), with a total of 2 arms. The studies found that patients using Sotagliflozin spent more time within the normal glucose range compared to the control group [8.53%, *95% CI*: 5.53 to 11.53, *P* < 0.05] (*I^2^ =* 45.1, *P*>0.05). Among these, the group using 400 mg of Sotagliflozin showed a further trend of increased time within the normal glucose range [10.67%, *95% CI*: 6.78 to 14.55, *P* < 0.05]. In addition, A total of 2 studies reported the time spent with glucose levels <3.9 mmol/L (12, 16), with a total of 2 arms. The studies found no statistical difference between the two groups in the time spent with glucose levels <3.9 mmol/L [-0.06%, 95% CI: -0.33 to 0.22, P > 0.05] and <3.0 mmol/L [-0.07%, 95% CI: -0.21 to 0.07, P > 0.05]. A total of 1 study (16), with 1 arm, found that patients using Sotagliflozin had significantly less time with blood glucose >10.0 mmol/L [-8.44%, *95% CI*: -15.10 to -1.77, *P* < 0.05] and >13.9 mmol/L [-1.40%, *95% CI*: -2.38 to -0.42, *P* < 0.05] compared to the control group. These results indicate that patients using Sotagliflozin spent significantly more time in the normal glucose range, while the frequency of hyperglycemic events was lower compared to the control group.

### 3.8 Other results

**Figure 7:**
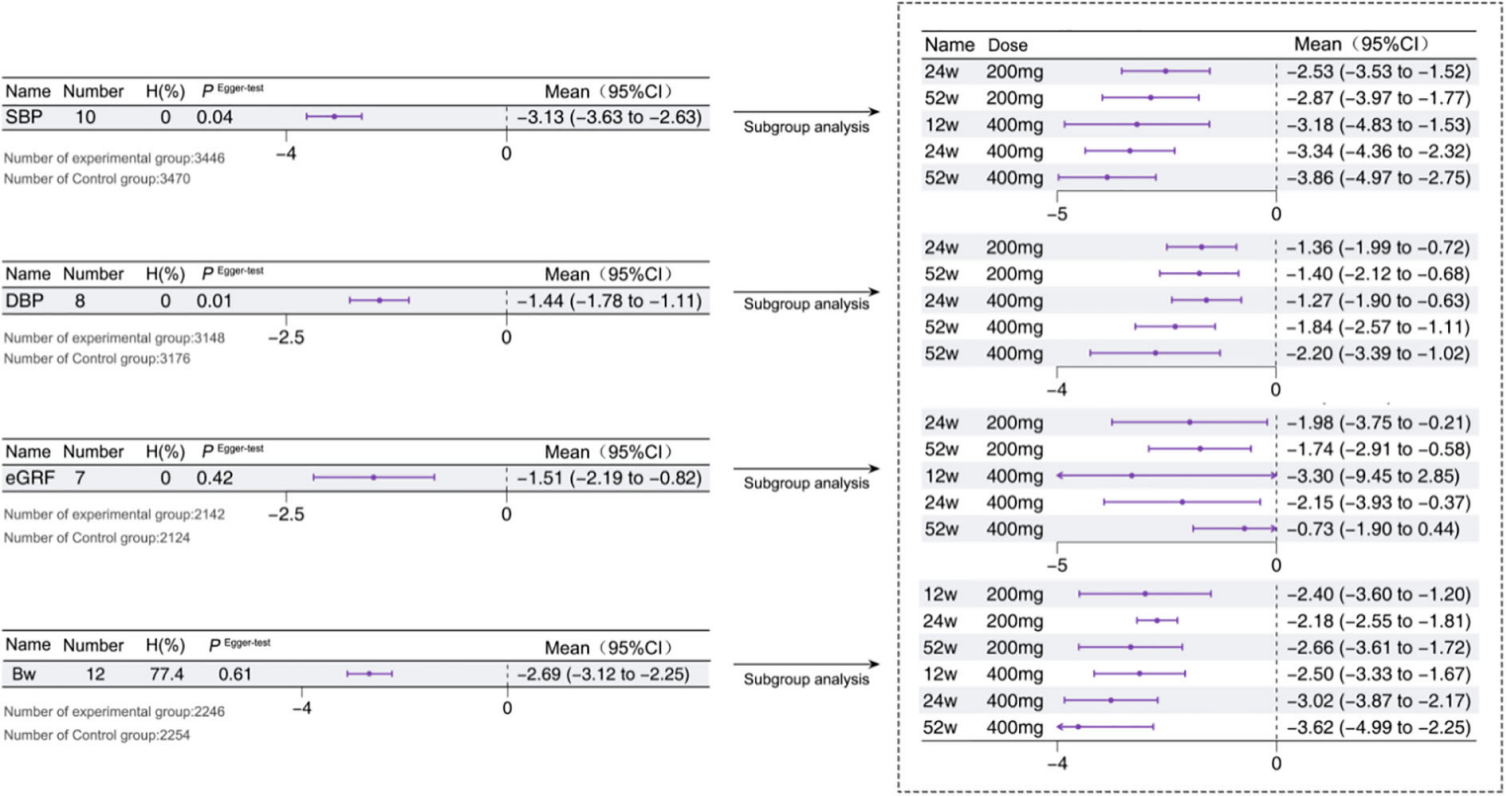
Other outcomes in T1D patients treated with adjunctive therapy using Sotagliflozin.

The extent of blood pressure reduction showed a positive correlation with both the intervention duration and drug concentration. A total of 5 studies reported on SBP (13–15, 18, 19), with a total of 10 arms. The studies indicated that combining Sotagliflozin with insulin therapy led to a significant reduction in SBP [-3.33 mmHg, *95% CI*: -3.13 to -2.63, *P* < 0.05] (*I^2^ =* 0.0, *P*>0.05). However, potential publication bias was detected in the Egger’s test, which was subsequently addressed using the cut-and-complement method, yielding revised results of [-3.00 mmHg, *95% CI*: -3.48 to -2.52, *P* < 0.05]. Additionally, a total of 3 studies reported the DBP (15, 18, 19), with a total of 8 arms. The studies indicated that combining Sotagliflozin with insulin therapy led to a significant reduction in DBP [-1.44 mmHg, *95% CI*: -1.78 to -1.11, *P* < 0.05] (*I^2^ =* 0.0, *P*>0.05). Similarly, potential publication bias was suggested by the Egger’s test, which was addressed using the cut-and-complement method, yielding revised results of [-1.24 mmHg, *95% CI*: -1.58 to -0.91, *P* < 0.05]. Furthermore, a total of 3 studies reported the eGFR, with a total of 7 arms. The analysis showed that the combination of Sotagliflozin and insulin therapy resulted in a reduction in eGFR [-1.51 mL/min/1.73m², *95% CI*: -2.19 to -0.82, *P* < 0.05] (*I^2^ =* 0.0, *P*>0.05). Moreover, a total of 4 studies reported the body weight (Bw) (12–15), with a total of 12 arms. The analysis revealed a significant reduction in Bw [-2.69 kg, *95% CI*: -3.13 to -2.63, *P* < 0.05] (*I^2^ =* 77.4, *P*<0.05). Notably, the difference in Bw between the experimental and control groups increased progressively with both the intervention duration and drug concentration, as shown in **Figure 7**.

### 3.9 Adverse effects

**Figure 8:**
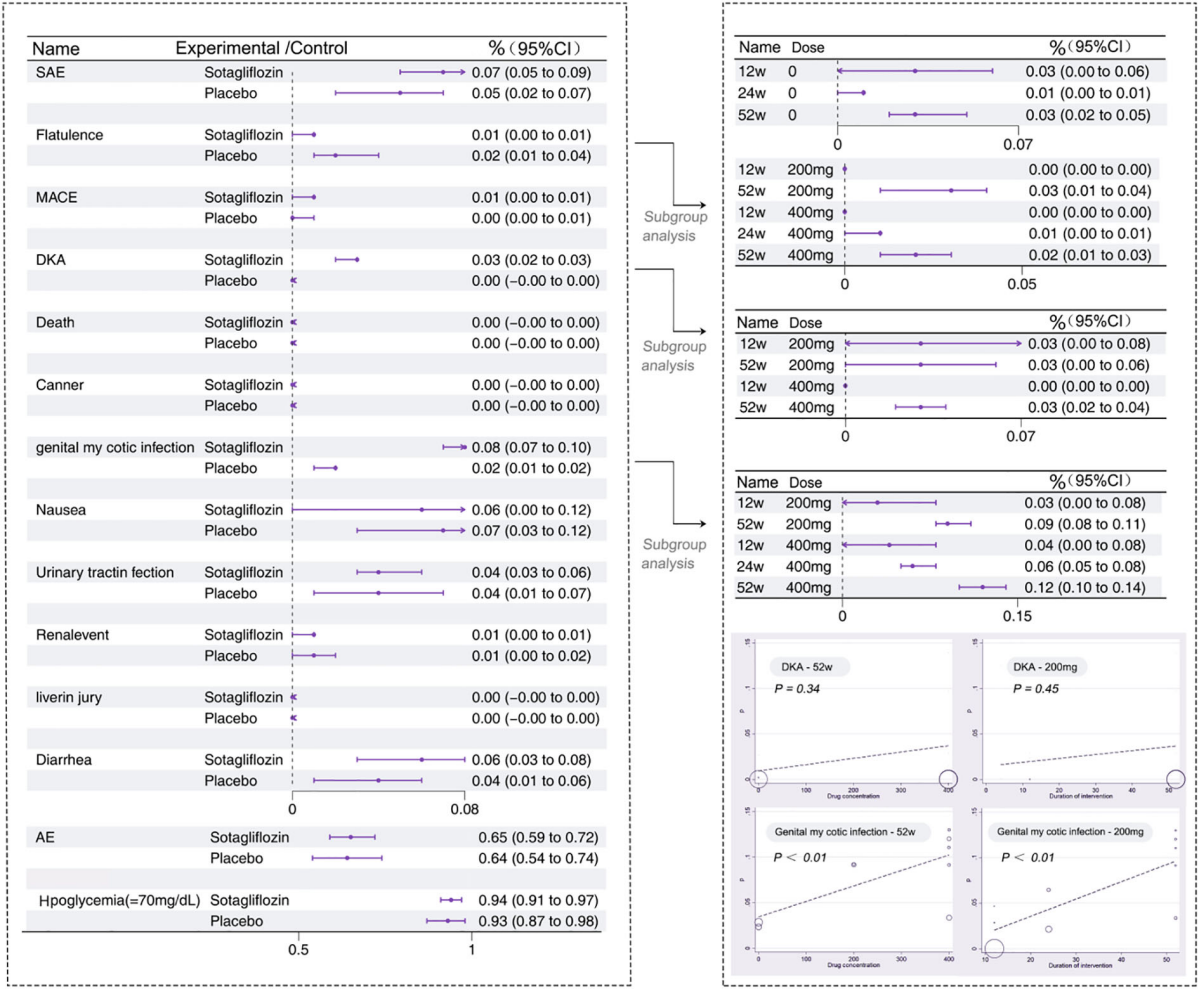
Summary graph of adverse effect analysis.

This meta-analysis explored the potential adverse effects of Sotagliflozin (12–15, 17, 20–22). A total of 7 studies reported adverse events (AE) (12–15, 17, 21, 22), with a total of 19 arms. The results indicated no statistically significant difference in the occurrence of AE[65%, *95% CI*: 59% to 72%, *P* < 0.05] (*I^2^ =* 92.4%, *P*<0.05) or SAE (12–15, 21, 22)[7%, *95% CI*: 5% to 9%, *P* < 0.05] (*I^2^ =* 89.4%, *P*<0.05) between the two groups. However, a total of 7 studies reported incidence of DKA (12–15, 17, 20, 22), with a total of 20 arms. The results indicated that the experimental group had a significantly higher incidence of DKA [3%, *95% CI*: 2% to 3%, *P* < 0.05] (*I^2^ =* 99.1% *P*<0.05) compared to the control group [0%, *95% CI*: 0% to 0%, *P* < 0.05] (*I^2^ =* 0.0, *P*>0.05). Notably, meta-regression analysis showed that the incidence of DKA was independent of both drug concentration and intervention duration, as depicted in **Figure 8**. The Egger’s test suggested a potential publication bias (*P* < 0.05), prompting recalibration of the DKA incidence using the cut-and-patch method, which yielded an adjusted incidence of 0.00% [95% CI: -0.01 to 0.01]. This adjustment revealed no statistically significant difference in DKA incidence between the experimental and control groups. Additionally, a total of 6 studies reported incidence of genital infections (12–15, 21, 22), with a total of 20 arms. The results indicated a significant difference in the incidence of genital infections, with the experimental group [8%, *95% CI*: 7% to 10%, *P* < 0.05] (*I^2^ =* 85.0, *P*<0.05) showing a higher rate than the control group [2%, *95% CI*: 1% to 2%, *P* < 0.05] (*I^2^ =* 0.0, *P*>0.05). Meta-regression analysis indicated a positive correlation between the infection rate and both drug concentration and intervention duration. A total of 6 studies reported incidence of genital infections (12–15, 17, 21), with a total of 16 arms. The study also suggested a potential reduction in fracture incidence in the experimental group (1% compared to 2% in the control group). Furthermore, when the drug concentration ranged between 200-400 mg/day, a higher drug concentration was significantly associated with an enhanced fracture-delaying effect. Specific adverse effects are visually summarized in **Figure 8**.

## 4 Discussion

**Figure 9:**
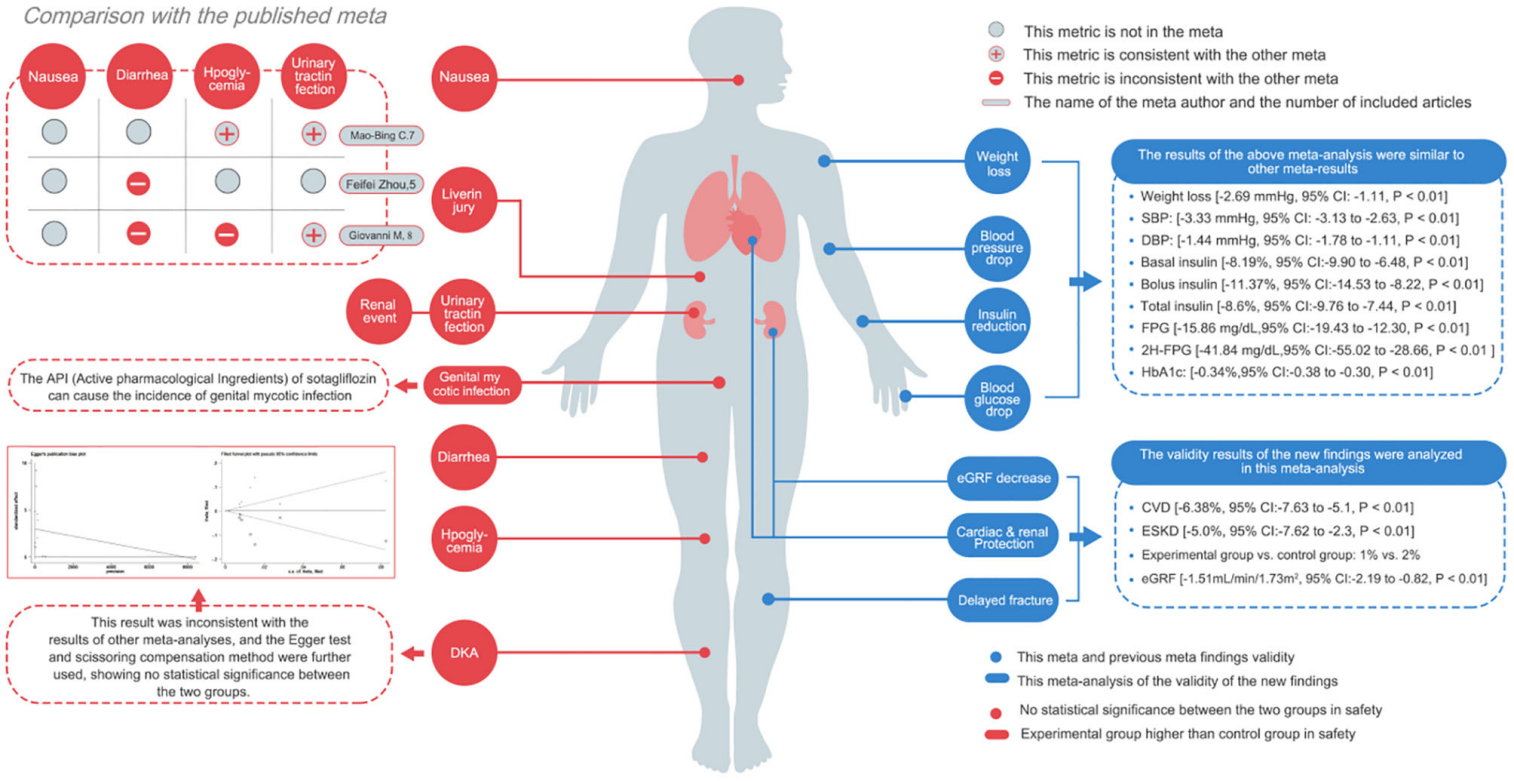
Comprehensive comparison with previously published meta-analyses.

Diabetes mellitus is a significant global endocrine disorder, posing a considerable threat to human health. Sotagliflozin, a dual inhibitor of SGLT1 and SGLT2, offers a distinct profile compared to traditional SGLT2 inhibitors (23–25). It not only reduces hepatic β-oxidation but also enhances the buffering capacity of the acid-base homeostasis system (26–28). By inhibiting SGLT1, Sotagliflozin reduces glucose entry into the bloodstream, contributing to better long-term control of 2H-PPG, lower glucagon production, and a decreased risk of cardiovascular disease. These mechanisms suggest that Sotagliflozin holds significant potential in improving the safety and efficacy of treatments for individuals with T1D. In this study, Sotagliflozin treatment resulted in a reduction in the 10-year incidence of cardiovascular disease (CVD) [-6.38%, *95% CI*: -7.63 to -5.1, *P* < 0.05] and ESKD [-5.0%, *95% CI*: -7.62 to -2.3, *P* < 0.05]. These reductions are likely due to improved glycemic and blood pressure control, further mitigating the risk of complications. Notably, when Sotagliflozin was administered in doses ranging from 200 mg to 400 mg per day, there was a more substantial decrease in blood glucose with increasing drug concentration. However, as the intervention duration extended from 12 to 52 weeks, the effect on glycemia showed a declining trend, possibly due to a reduction in insulin dosage. Despite this, the experimental group continued to show superior blood glycemia compared to the control group, with no significant difference in the incidence of hypoglycemic events. Additionally, the study demonstrated a modest reduction in blood pressure due to Sotagliflozin use, although the change was not of substantial magnitude [SBP: -3.33 mmHg, *95% CI*: -3.13 to -2.63, *P* < 0.05; DBP: -1.44 mmHg, *95% CI*: -1.78 to -1.11, *P* < 0.05]. This reduction was potentially associated with weight loss [-2.69 kg, *95% CI*: -3.13 to -2.63, *P* < 0.05], suggesting the need for further exploration of the antihypertensive effect through subgroup analyses involving body weight. In terms of adverse effects, Sotagliflozin did not increase the risk of urinary tract infections, consistent with previous studies. However, it did elevate the risk of genital infections, likely due to its pharmacological mechanism. Notably, genital mycotic infections were primarily observed in elderly patients within the first 30 days of treatment initiation. Interestingly, this study did not observe an increased risk of DKA, which contrasts with findings from previous meta-analyses. This discrepancy was investigated through Egger’s test and meta-regression, which indicated potential publication bias. After adjusting for this bias using the cut-and-patch method, the results suggested that Sotagliflozin did not increase the risk of DKA, though additional confirmation via RCTs may be needed. The study also indicated a potential reduction in fracture incidence, though the 95% CI showed some overlap for this outcome. Subgroup analyses suggested a clear trend, particularly relevant for osteoporosis prevention in middle-aged patients. Furthermore, other adverse effects, including nausea, diarrhea, liver injury, renal impairments, and cancer, showed no statistically significant differences between the experimental and control groups. A comprehensive comparison with previously published meta-analyses is available in **Figure 9** (29, 30).

This study found that Sotagliflozin can reduce the risk of CVD. On one hand, this may be attributed to its ability to more effectively control traditional risk factors such as weight, blood glucose, and blood pressure. The evidence from this study’s evidence-based approach supports this view.

1) Weight Reduction: Sotagliflozin induces weight loss by inhibiting SGLT1 and SGLT2 in both the kidneys and the intestines, leading to increased renal glucose excretion. As glucose is excreted, water and sodium are also eliminated from the body. The significant excretion of glucose requires additional energy to process, which promotes fat loss and helps reduce body weight. Furthermore, Sotagliflozin improves insulin sensitivity, reduces insulin resistance, and stimulates fat metabolism. However, P.C. Lee et al. found that the effect of SGLT inhibition on weight reduction is moderate and diminishes over time, which may be partly due to compensatory mechanisms, such as an increase in energy intake, which attempt to maintain body weight (31).

2) Blood Glucose Reduction: As an SGLT1/2 inhibitor, Sotagliflozin can more effectively lower blood glucose levels in patients with T1D compared to a placebo when used as an adjunctive therapy (8). This meta-analysis found that Sotagliflozin helps stabilize blood glucose and increase time within the normal glucose range, primarily by reducing hyperglycemia. Its effect relies on renal and intestinal glucose excretion rather than insulin secretion, which reduces the risk of hypoglycemia, especially at lower glucose levels. Additionally, in T1D patients, Sotagliflozin reduces beta-cell stress, facilitating better blood glycemia, lowering hyperglycemia risk, and extending time spent within the normal glucose range (23). At the same time, Sotagliflozin may offer renal protection by reducing the activity of renal SGLT2, potentially slowing the progression of kidney damage induced by diabetes. This, in turn, could indirectly lower the cardiovascular risk associated with diabetes. However, some studies suggest that hyperglycemia is a relatively weak risk factor for cardiovascular diseases, and that merely controlling blood glucose may not be directly linked to the risk of cardiovascular events (32, 33).

3) Blood Pressure Reduction: The exact mechanism behind the antihypertensive effects of SGLT inhibitors is not fully understood, but it may be mediated by the osmotic and diuretic effects of SGLT2 inhibitors, which inhibit sodium reabsorption in the proximal renal tubules. Inhibition of SGLT2 can lead to an approximately 50% increase in urinary sodium excretion (34). Additionally, SGLT inhibition may reduce sympathetic nervous system activity, inhibit norepinephrine conversion in brown adipose tissue, and decrease the production of tyrosine hydroxylase (35). However, the blood pressure-lowering effect of SGLT2 inhibitors is moderate. Moreover, compared to other cardiovascular diseases, the impact of blood pressure reduction on stroke incidence is more pronounced. Therefore, the role of Sotagliflozin in reducing cardiovascular risk through blood pressure control remains limited (36). On the other hand, Sotagliflozin may reduce the risk of CVD through mechanisms such as improving cardiac energy metabolism, reducing oxidative stress, and protecting endothelial cells.

4) Improvement of cardiac energy metabolism: Sotagliflozin can increase circulating ketone levels, which results from the mobilization of fatty acids from adipose tissue. These fatty acids are then utilized by the liver for ketogenesis. The resulting ketone compounds provide an enhanced energy supply to the heart (37). Simultaneously, Sotagliflozin promotes autophagy and lysosomal degradation, which improves mitochondrial morphology and function. These mitochondrial changes are beneficial for the heart’s energy supply. However, this enhanced energy supply does not necessarily correlate with improved efficiency of energy utilization by the heart (38). Additionally, SGLT1/2 inhibitors are associated with a reduction in the activity of calmodulin-dependent protein kinase II, which improves sarcoplasmic reticulum Ca^2+^ flux and increases cardiac contractility. This process may support cardiac energy conversion and help reduce the risk of CVD (39).

5) Reduction of oxidative stress and inflammatory response: Several studies have suggested that SGLT inhibitors can improve the inflammatory profile in patients with diabetes (40), potentially through extracellular matrix turnover and reduced fibrosis. Tsung-Ming Lee et al. found that Dapagliflozin exhibited significant antifibrotic effects by inhibiting collagen synthesis, thereby reducing the risk of cardiac remodeling. Moreover, the inhibition of SGLT1 in the heart may decrease myocardial sodium and glucose uptake, thereby reducing the generation of reactive oxygen species (ROS) induced by hyperglycemia (41, 42). However, some studies have indicated that dual SGLT1/2 inhibitors might exacerbate myocardial dysfunction in rats following myocardial infarction. Therefore, further investigation is required to assess the safety of Sotagliflozin in certain cardiovascular conditions (43).

6) Protection of endothelial cells: Studies have demonstrated that SGLT inhibition can improve vascular function by reducing endothelial cell activation, promoting direct vasodilation, alleviating endothelial dysfunction, and mitigating molecular changes associated with early atherosclerosis. These effects lead to decreased arterial stiffness and reduced total peripheral resistance (44). In this process, the inhibition of inflammatory pathways and the enhancement of mitochondrial function play crucial mediatory roles. Additionally, it has been proposed that SGLT2 inhibitors induce vasodilation through the activation of protein kinase G and voltage-gated potassium channels (45).

Regarding safety: This study found that Sotagliflozin does not increase the risk of fractures; rather, it appears to reduce the fracture risk, which may be linked to improved blood glycemia. As an adjunctive therapy, Sotagliflozin effectively lowers blood glucose levels, which helps alleviate diabetes-induced bone metabolism disorders, restore calcium and phosphate balance, and reduce skeletal damage caused by metabolic disturbances. The study also observed that, over the same follow-up period, the fracture risk associated with high-dose Sotagliflozin was lower than that associated with the low-dose regimen. Interestingly, while Sotaglifloxin did not increase the risk of urinary tract infections in our meta-analysis, we found that it elevated the risk of genital infections, particularly those caused by fungal pathogens, especially in elderly patients. Genital fungal infections predominantly occur within the first 30 days of treatment, and this phenomenon is also observed in patients with T2D (46). However, the exact mechanism remains unclear. It is likely related to the drug’s unique pharmacological action. The active ingredients in Sotagliflozin may increase the incidence of genital fungal infections in diabetic patients, possibly due to altered glucose metabolism and changes in the local immune response in the genital area (47). Additionally, SGLT1/2 inhibitors reduce renal glucose reabsorption, leading to glucosuria (48), as the concentration of glucose in the urinary environment rises, *Candida albicans*, the primary pathogen in diabetic patients, proliferates rapidly, contributing to the development of genital fungal infections. Furthermore, the presence of *Candida albicans* is also associated with impaired immune function in patients. Since T1D is an autoimmune disease, studies suggest that genital microbiome dysbiosis induced by autoimmune imbalance, combined with environmental changes caused by Sotagliflozin, may further increase the risk of genital fungal infections during T1D treatment (44). Moreover, Nyirjesy et al. suggested that the use of antifungal creams as adjunctive therapy can effectively prevent genital fungal infections (45).

DKA, one of the most severe complications of diabetes, has long been a topic of concern. Current research findings suggest that Sotagliflozin may increase the risk of DKA. However, this result may be influenced by publication bias. After adjusting the data using the trim and fill method, we found that Sotaglifloxin did not significantly increase the risk for DKA. Therefore, the relationship between Sotaglifloxin and DKA remains uncertain and requires further high-quality clinical studies. Traditionally, it is believed that SGLT2 inhibitors induce DKA primarily through the following mechanisms: 1) SGLT2 inhibitors predominantly act on the kidneys, leading to substantial glucose excretion via urine, which increases the risk of urinary tract infections (UTIs) and may subsequently induce DKA. However, Sotagliflozin, acting on both the kidneys and the small intestine epithelium, reduces the glucose load entering the bloodstream, thus potentially lowering the risk of urinary tract infections. This viewpoint is supported by the current meta-analysis, which shows no significant difference in the incidence of urinary tract infections between patients using Sotagliflozin and those on placebo (46). 2) In patients with T1D, insufficient insulin secretion, particularly after meals, leads to rapid exacerbation of hyperglycemia. This, in turn, increases the burden on the pancreas, promoting fatty acid oxidation and resulting in excessive ketone body production, potentially triggering DKA (47). Additionally, SGLT2 inhibitors cause continuous excretion of glucose by the kidneys, rapidly depleting endogenous glucose stores. In response, the body may break down fat to produce ketone bodies, thus maintaining energy supply (47). However, compared to SGLT2 inhibitors, Sotagliflozin has an advantage: its action on the intestinal epithelium reduces the rate at which glucose enters the bloodstream, thereby lowering insulin demand and preventing excessive fatty acid oxidation. Furthermore, as Sotagliflozin’s effect on the kidneys is weaker than that of pure SGLT2 inhibitors, this allows more time for glucagon secretion, which reduces fat breakdown and ketone body production. 3) Traditional SGLT2 inhibitors increase hepatic β-oxidation, leading to a rise in ketone body production and a reduction in bicarbonate production. In contrast, Sotagliflozin may attenuate hepatic β-oxidation, thereby enhancing the buffering capacity of the acid-base system and reducing the risk of DKA (48).

Limitations of the Study: 1) This study is limited by the lack of follow-up data, which prevents the exploration of long-term patient outcomes. Additionally, the relatively small number of included articles may introduce potential bias, highlighting the need for more high-quality RCTs. 2) The study also failed to establish a clear dose-response relationship, limiting the ability to quantitatively assess the drug’s safety and efficacy via response curves. Furthermore, the maximum observation period across the included studies was 52 weeks, which may not account for late-occurring adverse events such as major adverse cardiovascular events (MACE), mortality, or cancer. Future drug-targeted Mendelian randomized studies could be beneficial in further investigating and refining Sotagliflozin’s safety and efficacy. 3) Although this study included 12 articles, some were *post-hoc* analyses, meaning the actual number of clinical studies is lower than the number of articles included. More clinical RCTs are needed in the future to strengthen the evidence level.

## 5 Conclusion

In T1D patients, Sotagliflozin adjunct therapy improves blood glycemia, stabilizes blood pressure, and reduces cardiovascular risk factors. It also shows potential in lowering fracture risk, but the risk of DKA requires further clinical validation.

## Data Availability

The original contributions presented in the study are included in the article/**Supplementary Material**. Further inquiries can be directed to the corresponding authors.
