# Supplementary material for: Safety and efficacy of adjuvant Sotagliflozin therapy in patients with T1D - an update and systematic review and meta-analysis
Source: Front Endocrinol (Lausanne). 2025 Jun 3;16:1506652. doi: 10.3389/fendo.2025.1506652 (PMC12170572; doi:10.3389/fendo.2025.1506652)
Supplement: Supplementary file 1 [file DataSheet1.docx]

Table 1：Abbreviated situation table

| Full title | Abbreviation |
| --- | --- |
| Type 1 diabetes | T1D |
| Cardiovascular | CVD |
| End-stage kidney disease | ESKD |
| Fasting blood glucose | FPG |
| 2H postprandial blood glucose | 2H-FPG |
| Glycosylated hemoglobin | HbA1c |
| Systolic blood pressure | SBP |
| Diastolic blood pressure | DBP |
| Glomerular filtration rate | eGRF |
| Body weight | Bw |
| Severe adverse reactions | SAE |
| Adverse reactions | AE |
| Ketoacidosis | DKA |
| Major adverse cardiovascular events | Mace |

Table 2：Search formula

| Database | Search Strategy |
| --- | --- |
| PubMed (7) | ((((((((((((((((((((((((((((Diabetes Mellitus, Type 1[Title/Abstract]) OR (Diabetes Mellitus, Insulin-Dependent[Title/Abstract])) OR (Diabetes Mellitus, Insulin Dependent[Title/Abstract])) OR (Insulin-Dependent Diabetes Mellitus[Title/Abstract])) OR (Diabetes Mellitus, Juvenile-Onset[Title/Abstract])) OR (Diabetes Mellitus, Juvenile Onset[Title/Abstract])) OR (Juvenile-Onset Diabetes Mellitus[Title/Abstract])) OR (IDDM[Title/Abstract])) OR (Juvenile-Onset Diabetes[Title/Abstract])) OR (Diabetes, Juvenile-Onset[Title/Abstract])) OR (Juvenile Onset Diabetes[Title/Abstract])) OR (Diabetes Mellitus, Sudden-Onset[Title/Abstract])) OR (Diabetes Mellitus, Sudden Onset[Title/Abstract])) OR (Sudden-Onset Diabetes Mellitus[Title/Abstract])) OR (Type 1 Diabetes Mellitus[Title/Abstract])) OR (Diabetes Mellitus, Insulin-Dependent, 1[Title/Abstract])) OR (Insulin-Dependent Diabetes Mellitus 1[Title/Abstract])) OR (Insulin Dependent Diabetes Mellitus 1[Title/Abstract])) OR (Type 1 Diabetes[Title/Abstract])) OR (Diabetes, Type 1[Title/Abstract])) OR (Diabetes Mellitus, Type I[Title/Abstract])) OR (Diabetes, Autoimmune[Title/Abstract])) OR (Autoimmune Diabetes[Title/Abstract])) OR (Diabetes Mellitus, Brittle[Title/Abstract])) OR (Brittle Diabetes Mellitus[Title/Abstract])) OR (Diabetes Mellitus, Ketosis-Prone[Title/Abstract])) OR (Ketosis-Prone Diabetes Mellitus[Title/Abstract])) AND (((((((((((((((Sotagliflozin[Title/Abstract]) OR (Canagliflozin[Title/Abstract])) OR (Sodium Glucose Transporter 1[Title/Abstract])) OR (Sodium-Glucose Cotransporter 1[Title/Abstract])) OR (Sodium Glucose Cotransporter 1[Title/Abstract])) OR (SGLT1 Protein[Title/Abstract])) OR (Sodium Glucose Transporter 2 Inhibitors[Title/Abstract])) OR (SGLT-2 Inhibitors[Title/Abstract])) OR (Sodium-Glucose Transporter 2 Inhibitor[Title/Abstract])) OR (Sodium Glucose Transporter 2 Inhibitor[Title/Abstract])) OR (SGLT2 Inhibitor[Title/Abstract])) OR (Inhibitor, SGLT2[Title/Abstract])) OR (SGLT-2 Inhibitor[Title/Abstract])) OR (Inhibitor, SGLT-2[Title/Abstract])) OR (SGLT 2 Inhibitor[Title/Abstract]))) AND (((((Randomized control[Title/Abstract]) OR (Randomized controlled trial[Title/Abstract])) OR (Randomized study[Title/Abstract])) OR (RCT[Title/Abstract])) OR (Randomized studies[Title/Abstract])) |
| Embase (9) | #1:'diabetes mellitus, type 1':ab,ti OR 'diabetes mellitus, insulin-dependent':ab,ti OR 'diabetes mellitus, insulin dependent':ab,ti OR 'insulin-dependent diabetes mellitus':ab,ti OR 'diabetes mellitus, juvenile-onset':ab,ti OR 'diabetes mellitus, juvenile onset':ab,ti OR 'juvenile-onset diabetes mellitus':ab,ti OR 'iddm':ab,ti OR 'juvenile-onset diabetes':ab,ti OR 'diabetes, juvenile-onset':ab,ti OR 'juvenile onset diabetes':ab,ti OR 'diabetes mellitus, sudden-onset':ab,ti OR 'diabetes mellitus, sudden onset':ab,ti OR 'sudden-onset diabetes mellitus':ab,ti OR 'type 1 diabetes mellitus':ab,ti OR 'diabetes mellitus, insulin-dependent, 1':ab,ti OR 'insulin-dependent diabetes mellitus 1':ab,ti OR 'insulin dependent diabetes mellitus 1':ab,ti OR 'type 1 diabetes':ab,ti OR 'diabetes, type 1':ab,ti OR 'diabetes mellitus, type i':ab,ti OR 'diabetes, autoimmune':ab,ti OR 'autoimmune diabetes':ab,ti OR 'diabetes mellitus, brittle':ab,ti OR 'brittle diabetes mellitus':ab,ti OR 'diabetes mellitus, ketosis-prone':ab,ti OR 'ketosis-prone diabetes mellitus':ab,ti  #2:'sotagliflozin':ab,ti OR 'canagliflozin':ab,ti OR 'sodium glucose transporter 1':ab,ti OR 'sodium-glucose cotransporter 1':ab,ti OR 'sodium glucose cotransporter 1':ab,ti OR 'sglt1 protein':ab,ti OR 'sodium glucose transporter 2 inhibitors':ab,ti OR 'sglt-2 inhibitors':ab,ti OR 'sodium-glucose transporter 2 inhibitor':ab,ti OR 'sodium glucose transporter 2 inhibitor':ab,ti OR 'sglt2 inhibitor':ab,ti OR 'inhibitor, sglt2':ab,ti OR 'sglt-2 inhibitor':ab,ti OR 'inhibitor, sglt-2':ab,ti OR 'sglt 2 inhibitor':ab,ti  #3:'randomized control':ab,ti OR 'randomized controlled trial':ab,ti OR 'randomized study':ab,ti OR 'rct':ab,ti OR 'randomized studies':ab,ti  #1 AND #2 AND #3 |
| Cochrane (1734) | #1(Diabetes Mellitus, Type 1):ti,ab,kw OR (Diabetes Mellitus, Insulin-Dependent):ti,ab,kw OR (Diabetes Mellitus, Insulin Dependent):ti,ab,kw OR (Insulin-Dependent Diabetes Mellitus):ti,ab,kw OR (Diabetes Mellitus, Juvenile-Onset):ti,ab,kw OR (Diabetes Melli  #2 (Sotagliflozin):ti,ab,kw OR (Canagliflozin):ti,ab,kw OR (Sodium Glucose Transporter 1):ti,ab,kw OR (Sodium-Glucose Cotransporter 1):ti,ab,kw OR (Sodium Glucose Cotransporter 1):ti,ab,kw OR (SGLT1 Protein):ti,ab,kw OR (Sodium Glucose Transporter 2 Inhibitor  #3(Randomized control):ti,ab,kw OR (Randomized controlled trial):ti,ab,kw OR (Randomized study):ti,ab,kw OR (RCT):ti,ab,kw OR (Randomized studies):ti,ab,kw  #1 AND #2 AND #3 |
| Web of Science (1284) | **((((((((((((((((((((((((((TS=(Diabetes Mellitus, Type 1)) OR TS=(Diabetes Mellitus, Insulin-Dependent)) OR TS=(Diabetes Mellitus, Insulin Dependent)) OR TS=(Insulin-Dependent Diabetes Mellitus)) OR TS=(Diabetes Mellitus, Juvenile-Onset)) OR TS=(Diabetes Mellitus, Juvenile Onset)) OR TS=(Juvenile-Onset Diabetes Mellitus)) OR TS=(IDDM)) OR TS=(Juvenile-Onset Diabetes)) OR TS=(Diabetes, Juvenile-Onset)) OR TS=(Juvenile Onset Diabetes)) OR TS=(Diabetes Mellitus, Sudden-Onset)) OR TS=(Diabetes Mellitus, Sudden Onset)) OR TS=(Sudden-Onset Diabetes Mellitus)) OR TS=(Type 1 Diabetes Mellitus)) OR TS=(Diabetes Mellitus, Insulin-Dependent, 1)) OR TS=(Insulin-Dependent Diabetes Mellitus 1)) OR TS=(Insulin Dependent Diabetes Mellitus 1)) OR TS=(Type 1 Diabetes)) OR TS=(Diabetes, Type 1)) OR TS=(Diabetes Mellitus, Type I)) OR TS=(Diabetes, Autoimmune)) OR TS=(Autoimmune Diabetes)) OR TS=(Diabetes Mellitus, Brittle)) OR TS=(Brittle Diabetes Mellitus)) OR TS=(Diabetes Mellitus, Ketosis-Prone)) OR TS=(Ketosis-Prone Diabetes Mellitus)**  **((((((((((((((TS=(Sotagliflozin)) OR TS=(Canagliflozin)) OR TS=(Sodium Glucose Transporter 1)) OR TS=(Sodium-Glucose Cotransporter 1)) OR TS=(Sodium Glucose Cotransporter 1)) OR TS=(SGLT1 Protein)) OR TS=(Sodium Glucose Transporter 2 Inhibitors)) OR TS=(SGLT-2 Inhibitors)) OR TS=(Sodium-Glucose Transporter 2 Inhibitor)) OR TS=(Sodium Glucose Transporter 2 Inhibitor)) OR TS=(SGLT2 Inhibitor)) OR TS=(Inhibitor, SGLT2)) OR TS=(SGLT-2 Inhibitor)) OR TS=(Inhibitor, SGLT-2)) OR TS=(SGLT 2 Inhibitor)**  **((((TS=(Randomized control)) OR TS=(Randomized controlled trial)) OR TS=(Randomized study)) OR TS=(RCT)) OR TS=(Randomized studies)**  #1 AND #2 AND #3 |
| Scopus (3212) | ((TITLE-ABS-KEY(Diabetes Mellitus, Type 1) OR TITLE-ABS-KEY(Diabetes Mellitus, Insulin-Dependent) OR TITLE-ABS-KEY(Diabetes Mellitus, Insulin Dependent) OR TITLE-ABS-KEY(Insulin-Dependent Diabetes Mellitus) OR TITLE-ABS-KEY(Diabetes Mellitus, Juvenile-Onset) OR TITLE-ABS-KEY(Diabetes Mellitus, Juvenile Onset) OR TITLE-ABS-KEY(Juvenile-Onset Diabetes Mellitus) OR TITLE-ABS-KEY(IDDM) OR TITLE-ABS-KEY(Juvenile-Onset Diabetes) OR TITLE-ABS-KEY(Diabetes, Juvenile-Onset) OR TITLE-ABS-KEY(Juvenile Onset Diabetes) OR TITLE-ABS-KEY(Diabetes Mellitus, Sudden-Onset) OR TITLE-ABS-KEY(Diabetes Mellitus, Sudden Onset) OR TITLE-ABS-KEY(Sudden-Onset Diabetes Mellitus) OR TITLE-ABS-KEY(Type 1 Diabetes Mellitus) OR TITLE-ABS-KEY(Diabetes Mellitus, Insulin-Dependent, 1) OR TITLE-ABS-KEY(Insulin-Dependent Diabetes Mellitus 1) OR TITLE-ABS-KEY(Insulin Dependent Diabetes Mellitus 1) OR TITLE-ABS-KEY(Type 1 Diabetes) OR TITLE-ABS-KEY(Diabetes, Type 1) OR TITLE-ABS-KEY(Diabetes Mellitus, Type I) OR TITLE-ABS-KEY(Diabetes, Autoimmune) OR TITLE-ABS-KEY(Autoimmune Diabetes) OR TITLE-ABS-KEY(Diabetes Mellitus, Brittle) OR TITLE-ABS-KEY(Brittle Diabetes Mellitus) OR TITLE-ABS-KEY(Diabetes Mellitus, Ketosis-Prone) OR TITLE-ABS-KEY(Ketosis-Prone Diabetes Mellitus))) AND ((TITLE-ABS-KEY(Sotagliflozin) OR TITLE-ABS-KEY(Canagliflozin) OR TITLE-ABS-KEY(Sodium Glucose Transporter 1) OR TITLE-ABS-KEY(Sodium-Glucose Cotransporter 1) OR TITLE-ABS-KEY(Sodium Glucose Cotransporter 1) OR TITLE-ABS-KEY(SGLT1 Protein) OR TITLE-ABS-KEY(Sodium Glucose Transporter 2 Inhibitors) OR TITLE-ABS-KEY(SGLT-2 Inhibitors) OR TITLE-ABS-KEY(Sodium-Glucose Transporter 2 Inhibitor) OR TITLE-ABS-KEY(Sodium Glucose Transporter 2 Inhibitor) OR TITLE-ABS-KEY(SGLT2 Inhibitor) OR TITLE-ABS-KEY(Inhibitor, SGLT2) OR TITLE-ABS-KEY(SGLT-2 Inhibitor) OR TITLE-ABS-KEY(Inhibitor, SGLT-2) OR TITLE-ABS-KEY(SGLT 2 Inhibitor))) AND ((TITLE-ABS-KEY(Randomized control) OR TITLE-ABS-KEY(Randomized controlled trial) OR TITLE-ABS-KEY(Randomized study) OR TITLE-ABS-KEY(RCT) OR TITLE-ABS-KEY(Randomized studies))) |
