# Supplementary material for: Safety and efficacy of adjuvant Sotagliflozin therapy in patients with T1D - an update and systematic review and meta-analysis
Source: Front Endocrinol (Lausanne). 2025 Jun 3;16:1506652. doi: 10.3389/fendo.2025.1506652 (PMC12170572; doi:10.3389/fendo.2025.1506652)
Supplement: Supplementary file 2 [file DataSheet2.pdf]

# Table1. GRADE evidence-based level

| Quality assessment | | | | | | | No of patients | | Quality | Importance |
|---|---|---|---|---|---|---|---|---|---|---|
| No of studies | Design | Risk of bias | Inconsistency | Indirectness | Imprecision | Other considerations | Experimental group | Control group | | |
| **Flatulence** | | | | | | | | | | |
| 6 | randomised trials | no serious risk of bias | no serious inconsistency | no serious indirectness | no serious imprecision | reporting bias[1] | 32/1912 | 26/1324 | ⊕⊕⊕O | IMPORTANT |
| | | | | | | | 1.70% | 2% | MODERATE | |
| **MACE** | | | | | | | | | | |
| 3 | randomised trials | no serious risk of bias | no serious inconsistency | no serious indirectness | no serious imprecision | reporting bias | 15/1748 | 7/1229 | ⊕⊕⊕O | CRITICAL |
| | | | | | | | 0.86% | 0.57% | MODERATE | |
| **DKA** | | | | | | | | | | |
| 7 | randomised trials | no serious risk of bias | no serious inconsistency | no serious indirectness | no serious imprecision | reporting bias[1] | 175/3311 | 16/1673 | ⊕⊕⊕O | CRITICAL |
| | | | | | | | 5.30% | 0.96% | MODERATE | |
| **Death** | | | | | | | | | | |
| 5 | randomised trials | no serious risk of bias | no serious inconsistency | no serious indirectness | no serious imprecision | none | 1/1896 | 3/1307 | ⊕⊕⊕⊕ | CRITICAL |
| | | | | | | | 0.05% | 0.23% | HIGH | |
| **Cancer** | | | | | | | | | | |
| 4 | randomised trials | no serious risk of bias | no serious inconsistency | no serious indirectness | no serious imprecision | none | 7/1371 | 4/1039 | ⊕⊕⊕⊕ | CRITICAL |
| | | | | | | | 0.51% | 0.38% | HIGH | |

| | | | | | | | | | | |
|---|---|---|---|---|---|---|---|---|---|---|
| **Genital my cotic infection** | | | | | | | | | | |
| 6 | randomised trials | no serious risk of bias | no serious inconsistency | no serious indirectness | no serious imprecision | reporting bias | 325/3469<br>9.40% | 51/2091<br>2.40% | ⊕⊕⊕O<br>MODERATE | IMPORTANT |
| **Nausea** | | | | | | | | | | |
| 2 | randomised trials | no serious risk of bias | no serious inconsistency | no serious indirectness | no serious imprecision | reporting bias[1] | 9/118<br>7.60% | 9/118<br>7.60% | ⊕⊕⊕O<br>MODERATE | IMPORTANT |
| **Hypoglycemia** | | | | | | | | | | |
| 5 | randomised trials | no serious risk of bias | no serious inconsistency | no serious indirectness | no serious imprecision | reporting bias | 2232/2464<br>90.60% | 1436/1617<br>88.80% | ⊕⊕⊕O<br>MODERATE | IMPORTANT |
| **Liver injury** | | | | | | | | | | |
| 2 | randomised trials | no serious risk of bias | no serious inconsistency | no serious indirectness | no serious imprecision | reporting bias | 4/1224<br>0.33% | 0/971<br>0% | ⊕⊕⊕O<br>MODERATE | IMPORTANT |
| **Renal event** | | | | | | | | | | |
| 3 | randomised trials | no serious risk of bias | no serious inconsistency | no serious indirectness | no serious imprecision | reporting bias | 20/1748<br>1.10% | 11/1229<br>0.90% | ⊕⊕⊕O<br>MODERATE | IMPORTANT |
| **Urinary tract infection** | | | | | | | | | | |
| 6 | randomised trials | no serious risk of bias | no serious inconsistency | no serious indirectness | no serious imprecision | reporting bias | 160/2945<br>5.40% | 93/1883<br>4.90% | ⊕⊕⊕O<br>MODERATE | IMPORTANT |
| **Diarrhea** | | | | | | | | | | |
| 6 | randomised | no serious | no serious | no serious | no serious | none | 193/2945 | 73/1833 | ⊕⊕⊕⊕ | IMPORTANT |

| | | | | | | | | | | |
|---|---|---|---|---|---|---|---|---|---|---|
| | trials | risk of bias | inconsistency | indirectness | imprecision | | 6.60% | 4% | HIGH | |
| **10-year CVD risk** | | | | | | | | | | |
| 1 | randomised trials | no serious risk of bias | no serious inconsistency | no serious indirectness | no serious imprecision | reporting bias | 2246 | 2212 | ⊕⊕⊕O MODERATE | CRITICAL |
| **5-year CVD risk(%)** | | | | | | | | | | |
| 1 | randomised trials | no serious risk of bias | no serious inconsistency | no serious indirectness | no serious imprecision | reporting bias | 2115 | 2286 | ⊕⊕⊕O MODERATE | CRITICAL |
| **5-year ESKD risk(%)** | | | | | | | | | | |
| 1 | randomised trials | no serious risk of bias | no serious inconsistency | no serious indirectness | no serious imprecision | reporting bias | 1169 | 1659 | ⊕⊕⊕O MODERATE | CRITICAL |
| **A1C (%)** | | | | | | | | | | |
| 4 | randomised trials | no serious risk of bias | no serious inconsistency | no serious indirectness | no serious imprecision | none | 2246 | 2212 | ⊕⊕⊕⊕ HIGH | CRITICAL |
| **2-h PPG (mg/dL)** | | | | | | | | | | |
| 3 | randomised trials | no serious risk of bias | no serious inconsistency | no serious indirectness | no serious imprecision | none | 333 | 336 | ⊕⊕⊕⊕ HIGH | CRITICAL |
| **FPG (mg/dL)** | | | | | | | | | | |
| 4 | randomised trials | no serious risk of bias | no serious inconsistency | no serious indirectness | no serious imprecision | none | 2246 | 2254 | ⊕⊕⊕⊕ HIGH | CRITICAL |

| | | | | | | | | | | |
|---|---|---|---|---|---|---|---|---|---|---|
| **Basal insulin dose (%)** | | | | | | | | | | |
| 2 | randomised trials | no serious risk of bias | no serious inconsistency | no serious indirectness | no serious imprecision | none | 1093 | 1114 | ⊕⊕⊕⊕<br>HIGH | CRITICAL |
| **Bolus insulin dose (%)** | | | | | | | | | | |
| 3 | randomised trials | no serious risk of bias | no serious inconsistency | no serious indirectness | no serious imprecision | none | 1617 | 1630 | ⊕⊕⊕⊕<br>HIGH | CRITICAL |
| **Total daily insulin dose (%)** | | | | | | | | | | |
| 3 | randomised trials | no serious risk of bias | no serious inconsistency | no serious indirectness | no serious imprecision | none | 2141 | 2146 | ⊕⊕⊕⊕<br>HIGH | IMPORTANT |
| **Body weight (kg)** | | | | | | | | | | |
| 4 | randomised trials | no serious risk of bias | no serious inconsistency | no serious indirectness | no serious imprecision | none | 2246 | 2254 | ⊕⊕⊕⊕<br>HIGH | IMPORTANT |
| **SBP(mmgh)** | | | | | | | | | | |
| 5 | randomised trials | no serious risk of bias | no serious inconsistency | no serious indirectness | no serious imprecision | reporting bias | 5814 | 5864 | ⊕⊕⊕O<br>MODERATE | IMPORTANT |
| **DBP(mmgh)** | | | | | | | | | | |
| 3 | randomised trials | no serious risk of bias | no serious inconsistency | no serious indirectness | no serious imprecision | reporting bias | 4197 | 4228 | ⊕⊕⊕O<br>MODERATE | IMPORTANT |
| **eGFR (ml/min/1.73M2) (Better indicated by lower values)** | | | | | | | | | | |
| 3 | randomised | no serious | no serious | no serious | no serious | none | 2142 | 2166 | ⊕⊕⊕⊕ | IMPORTANT |

| | | | | | | | | | | |
|---|---|---|---|---|---|---|---|---|---|---|
| | trials | risk of bias | inconsistency | indirectness | imprecision | | | | HIGH | |

**Table1. The Egger test and the cut-and-patch method**

| Variable | Subgroups | *P*-Egger | Cut and fill method | | |
|---|---|---|---|---|---|
| | | | Effect size | 95CI% | |
| FPG (mg/dL) | - | 0.01 | -1.03 | -1.65 | -0.42 |
| 2H-FPG (mg/dL) | - | 0.57 | | | |
| HbA1c (%) | - | 0.99 | | | |
| Basal insulin dose (%) | - | 0.83 | | | |
| Bolus insulin dose (%) | - | 0.74 | | | |
| Total daily insulin dose (%) | - | 0.75 | | | |
| SBP (mmgh) | - | 0.04 | -3.13 | -4.13 | -2.12 |
| DBP (mmgh) | - | 0.02 | -1.44 | -2.12 | -0.77 |
| eGRF (mL/min/1.73M$^2$) | - | 0.42 | | | |
| Bw (kg) | - | 0.61 | | | |
| AnyAE | 200mg | 0.14 | | | |
| | 400mg | 0.78 | | | |
| | Placebo | 0.32 | | | |
| Cancer | 200mg | - | | | |
| | 400mg | 0.15 | | | |
| | Placebo | - | | | |
| Deaths | 200mg | - | | | |

| | | | | | |
|---|---|---|---|---|---|
| | 400mg | 0.06 | | | |
| | Placebo | - | | | |
| Diarrhea | 200mg | 0.18 | | | |
| | 400mg | 0.01 | 0.01 | -0.02 | 0.02 |
| | Placebo | 0.68 | | | |
| DKA | 200mg | 0.20 | | | |
| | 400mg | 0.02 | 0.00 | -0.01 | 0.01 |
| | Placebo | 0.27 | | | |
| Hypoglycemia | 200mg | - | | | |
| | 400mg | 0.04 | 0.97 | 0.96 | 0.98 |
| | Placebo | - | | | |
| Flatulence | 200mg | - | | | |
| | 400mg | 0.01 | 0.00 | 0.00 | 0.01 |
| | Placebo | - | | | |
| Genital my cotic infection | 200mg | 0.02 | 0.06 | 0.01 | 0.13 |
| | 400mg | 0.01 | 0.06 | 0.04 | 0.09 |
| | Placebo | - | | | |
| MACE | 200mg | - | | | |
| | 400mg | 0.01 | 0.00 | -0.01 | 0.01 |
| | Placebo | - | | | |
| Nausea | 200mg | - | | | |
| | 400mg | 0.01 | 0.04 | 0.01 | 0.08 |
| | Placebo | - | | | |
| Liver injury | 200mg | - | | | |
| | 400mg | 0.03 | 0.00 | -0.00 | 0.00 |

| | | | | | |
|---|---|---|---|---|---|
| | Placebo | - | | | |
| Renal event | 200mg | - | | | |
| | 400mg | 0.01 | 0.01 | 0.00 | 0.01 |
| | Placebo | - | | | |
| SAE | 200mg | 0.29 | | | |
| | 400mg | 0.01 | 0.06 | 0.04 | 0.08 |
| | Placebo | 0.16 | | | |
| Urinary tract infection | 200mg | 0.20 | | | |
| | 400mg | 0.01 | 0.01 | -0.01 | 0.02 |
| | Placebo | 0.79 | | | |
