# Supplementary material for: Safety and efficacy of adjuvant Sotagliflozin therapy in patients with T1D - an update and systematic review and meta-analysis
Source: Front Endocrinol (Lausanne). 2025 Jun 3;16:1506652. doi: 10.3389/fendo.2025.1506652 (PMC12170572; doi:10.3389/fendo.2025.1506652)
Supplement: Supplementary file 3 [file DataSheet3.docx]

| [Anne L Peters](https://pubmed.99885.net/?sort=date&term=Peters+AL&cauthor_id=32928957)^[20]^ | 2020 | 524 | 526 | Sotagliflozin 200 mg | 44.4 ± 13.7 | 42.5 ± 13.3 | 28.9 ± 5.6 | 28.5 ± 5.3 | 52w |
| --- | --- | --- | --- | --- | --- | --- | --- | --- | --- |
|  |  | 525 | 526 | Sotagliflozin 400 mg | 44.0 ± 13.4 | 42.5 ± 13.3 | 28.7 ± 5.2 | 28.5 ± 5.3 | 52w |
| [Claire Baker](https://pubmed.99885.net/?sort=date&term=Baker+C&cauthor_id=31264767)^[13]^ | 2019 | 35 | 36 | Sotagliflozin 75 mg | 42.4 ± 12.0 | 48.1 ± 11.3 | 27.4 ± 5.0 | 31.8 ± 5.8 | 12w |
|  |  | 35 | 36 | Sotagliflozin 200 mg | 47.0 ± 14.0 | 48.1 ± 11.3 | 28.0 ± 4.7 | 31.8 ± 5.8 | 12w |
|  |  | 35 | 36 | Sotagliflozin 400 mg | 44.8 ± 15.4 | 48.1 ± 11.3 | 29.4 ± 5.8 | 31.8 ± 5.8 | 12w |
|  |  | 524 | 526 | Sotagliflozin 200 mg | 44.4 ± 13.7 | 42.5 ± 13.3 | 28.9 ± 5.6 | 28.5 ± 5.3 | 52w |
| [Helena W Rodbard](https://pubmed.99885.net/?sort=date&term=Rodbard+HW&cauthor_id=33611925)^[18]^ | 2021 | 525 | 526 | Sotagliflozin 400 mg | 44.0 ± 13.4 | 42.5 ± 13.3 | 28.7 ± 5.2 | 28.5 ± 5.3 | 52w |
| [Satish K Garg](https://pubmed.99885.net/?sort=date&term=Garg+SK&cauthor_id=28899222)^[21]^ | 2017 | 703 | 699 | Sotagliflozin 400 mg | 43.3 ± 14.2 | 42.4 ± 14.0 | 28.29 ± 5.13 | 28.10 ± 5.18 | 24w |
| [Bruce W Bode](https://pubmed.99885.net/?sort=date&term=Bode+BW&cauthor_id=32640846)^[12]^ | 2021 | 43 | 42 | Sotagliflozin 400 mg | 22.8 ± 4.0 | 21.7 ± 3.6 | 29.4 ± 7.2 | 26.7 ± 5.0 | 12w |
| Thomas Danne^[14]^ | 2018 | 261 | 258 | Sotagliflozin 200 mg | Na | Na | Na | Na | 52w |
|  |  | 263 | 258 | Sotagliflozin 400 mg | Na | Na | Na | Na | 52w |
| [Thomas Danne](https://pubmed.ncbi.nlm.nih.gov/?term=Danne%20T%5bAuthor%5d" \o "https://pubmed.ncbi.nlm.nih.gov/?term=Danne T[Author])^[16]^ | 2019 | 89 | 93 | Sotagliflozin 200 mg | 44.2 ± 13.2 | 43.5 ± 14.2 | 29.5 ± 4.8 | 29.4 ± 5.5 | 24w |
|  |  | 96 | 93 | Sotagliflozin 400 mg | 45.1 ± 12.1 | 43.5 ± 14.2 | 29.7 ± 4.9 | 29.4 ± 5.5 | 24w |
|  |  | 524 | 526 | Sotagliflozin 200 mg | 44.4 ± 13.7 | 42.5 ± 13.3 | 28.9 ± 5.6 | 28.5 ± 5.3 | 52w |
| [Helena W Rodbard](https://pubmed.99885.net/?sort=date&term=Rodbard+HW&cauthor_id=32618383)^[22]^ | 2020 | 525 | 526 | Sotagliflozin 400 mg | 44.0 ± 13.4 | 42.5 ± 13.3 | 28.7 ± 5.2 | 28.5 ± 5.3 | 52w |
| John B Buse^[15]^ | 2018 | 263 | 268 | Sotagliflozin 200 mg | 46.6 ± 13.48 | 45.2 ± 12.72 | 29.81 ± 5.68 | 29.55 ± 5.18 | 52w |
|  |  | 263 | 268 | Sotagliflozin 400 mg | 46.4 ± 13.12 | 45.2 ± 12.72 | 29.63 ± 5.29 | 29.55 ± 5.18 | 52w |
| Arthur T Sands^[17]^ | 2015 | 16 | 17 | Sotagliflozin 400 mg | 45.5 ± 21.55 | 34.0 ± 21.57 | 27.1 ± 3.1 | 26.2 ± 3.0 | 4w |
|  |  | 524 | 526 | Sotagliflozin 200 mg | 44.4 ± 13.7 | 42.5 ± 13.3 | 28.9 ± 5.6 | 28.5 ± 5.3 | 52w |
| Daniël H. van Raalte^[19]^ | 2019 | 525 | 526 | Sotagliflozin 400 mg | 44.0 ± 13.4 | 42.5 ± 13.3 | 28.7 ± 5.2 | 28.5 ± 5.3 | 52w |
| [EB Stougaard](https://scholar.66557.net/citations?user=eWkPmqwAAAAJ&hl=zh-CN&newwindow=1&oi=sra)^[11]^ | 2023 | 1659 | 1169 | Sotagliflozin 200/400 mg | 43.8 ± 13.8 | 42.5 ± 13.7 | NA | NA | 24w |
| Author | Year | Number of subjects | Number of controls | Intervention | The experimental group age | The control group age | The experimental group BMI | The control group BMI | Duration of intervention |

NA: Numerical values are missing. All continuous numerical variables are expressed using Mean±SD.
